# Cognitive Electrophysiology in Socioeconomic Context in Adulthood

**DOI:** 10.1038/s41597-025-05209-z

**Published:** 2025-05-22

**Authors:** Elif Isbell, Amanda N. Peters, Dylan M. Richardson, Nancy E. Rodas De León

**Affiliations:** Department of Psychological Sciences, University of California Merced, Merced, CA 95343 USA

**Keywords:** Cognitive neuroscience, Human behaviour

## Abstract

This dataset contains electroencephalography (EEG) recordings of 127 young adults (18–30 years old), along with retrospective objective and subjective reports of childhood family socioeconomic status (SES), as well as SES indicators in adulthood, such as educational attainment, food security, and home and neighborhood characteristics. The EEG data were recorded during commonly used cognitive electrophysiology tasks that were directly acquired or adapted from the Event-Related Potentials Compendium of Open Resources and Experiments, i.e., ERP CORE. This dataset can be used to address questions of cognitive electrophysiology in the context of childhood and adulthood SES. It can also be used to conduct EEG methodology research, such as investigating the precision and reliability of measurements in diverse samples of young adults. In addition, this dataset includes self-reports of attention-deficit/hyperactivity disorder (ADHD) symptoms and can be used to assess the links between ADHD symptomatology and cognitive electrophysiology in young adults from diverse socioeconomic backgrounds. The dataset is available on OpenNeuro.

## Background & Summary

Electroencephalography (EEG) is a widely used neuroscience method for measuring the brain’s electrophysiological signals^1–4^. Capturing the electrochemical activity of large neural populations via sensors placed on a cap, EEG allows us to study neural responses that manifest within milliseconds of an event, such as the occurrence of stimuli or engagement in motor actions. This method has been widely used for decades, advancing our understanding of human perception, cognition, emotion, and action in neurotypical adults^4–6^. In addition, the excellent temporal resolution of EEG has allowed for the discovery of various divergences in *how* and *when* neural responses are observed during task engagement in clinical adult populations compared to neurotypical adults. Such divergences in brain electrophysiology have been implicated in a wide range of neurodevelopmental and mental health disorders, including schizophrenia, autism spectrum disorder, attention-deficit/hyperactivity disorder (ADHD), and depression^7–12^.

Despite its notable contributions and promise to advance our fundamental understanding of typical and divergent brain functioning, it has been argued that the majority of human cognition and neuroscience research included racially, ethnically, socioeconomically, and linguistically homogenous samples^13–15^. It remains challenging to evaluate this claim empirically due to a lack of information on sample characteristics. The vast majority of cognition and neuroscience research, especially experimental research with neurotypical adults, has not reported on the sociodemographic characteristics of their samples, beyond age range and sex, or the context in which the data were collected, such as reporting that the sample consisted of “university students”^13^.

To contribute to addressing these issues, here we present a cognitive electrophysiology dataset that includes various sociodemographic indicators, with a particular focus on indicators of childhood and adulthood socioeconomic context. Family socioeconomic status (SES), as measured by indicators such as parental education, income, occupation, and family wealth, has been associated with brain development and functioning in childhood^16–18^. As it pertains to cognitive electrophysiology, lower parental educational attainment and family income-to-needs ratio have been linked to divergent neural responses in childhood, such as attenuated neural responses when engagement of cognitive control is needed or larger neural responses to distracting stimuli^19–22^. A few longitudinal studies, as well as those that acquired retrospective accounts of childhood, also linked childhood family SES to brain functioning in adulthood^23–26^. In addition to childhood family SES, adults’ objective and subjective evaluations of their social status have also been linked to structural and functional brain characteristics from early to later years of adulthood^27–29^. Together, these studies underline socioeconomic context as an important contributor to brain development and functioning across the lifespan.

Building on this research, we collected EEG data from young adults, along with retrospective objective and subjective indicators of childhood family SES, as well as SES indicators in adulthood, such as educational attainment, food security, and home and neighborhood characteristics. The dataset we describe here includes EEG data collected from 127 young adults (18–30 years old) with diverse childhood family socioeconomic backgrounds. The EEG data were recorded with tasks that were directly acquired or adapted from the Event-Related Potentials Compendium of Open Resources and Experiments, i.e., ERP CORE^1^. These tasks, which are publicly available, were optimized to capture neural activity manifest in perception, cognition, and action in neurotypical young adults. Therefore, in addition to addressing questions about the links between childhood and adulthood socioeconomic context and cognitive electrophysiology, this dataset can be used for EEG methodology investigations, such as measuring the precision and reliability of EEG measures across datasets that used these paradigms. Furthermore, the dataset includes a symptoms checklist, comprised of questions that predicted symptoms consistent with ADHD in adulthood^30^, which can be used to investigate the links between ADHD symptoms and neural activity in a socioeconomically diverse young adult sample.

A subset of this dataset was previously included in a publication that examined the associations between objective and subjective indicators of childhood family SES and two well-established ERP indices of brain functioning in adulthood, captured in the passive auditory oddball and active visual oddball tasks^25^. The dataset described here features additional participants whose data were collected as part of other ongoing projects in our research lab that included childhood and adulthood SES indicators. Furthermore, this dataset includes EEG tasks and self-report measures which have not been included in any prior publication, comprising two additional EEG tasks from ERP CORE^1^ (a simple visual search and a flanker task), and self-report measures of adult socioeconomic indicators—including educational attainment, food security, home and neighborhood characteristics—as well as adult ADHD symptoms. The dataset provides full continuous EEG recordings for each task, providing opportunities for analyses beyond ERPs, such as spectral analyses and spectral parameterization approaches. Overall, the sharing of this dataset and accompanying materials will contribute to efforts toward reproducible cognitive neuroscience research that is embedded in individuals’ sociodemographic context.

## Method

### Participants

We recruited participants via flyers posted on a university campus and at community centers, businesses, and public spaces in Merced County, California, United States. To match the age range in the original ERP CORE study^1^, we recruited participants between the ages of 18 and 30 years. Individuals who met any of the following criteria were excluded from the study: hearing issues, history of brain injury or neurological disorders, absence of normal or corrected-to-normal vision, and current use of brain altering medications. The dataset comprises participants recruited for studies on adult cognition in context. To provide the largest sample size, we included all participants who completed the childhood and adulthood SES surveys and at least one of the EEG tasks of interest.

While this approach maximized the sample size per EEG task and increased the statistical power for analyses involving each EEG task, it also meant that some participants had the opportunity to complete certain tasks but not others due to the design of the ongoing projects. The missingness per task was as follows: For auditory oddball, 117 participants had the opportunity to complete the task. EEG data were missing for 3 participants due to equipment error (trigger code or headphone malfunction). For visual oddball, 100 participants had the opportunity to complete the task. EEG data were missing for 1 participant due to a participant-related issue (early departure). For flanker and visual search, 73 participants had the opportunity to complete the tasks. Across these participants, no EEG data were missing in the flanker task, and EEG data were missing for 1 participant in the visual search task (participant verbally disclosed color-blindness after the session started). The participants completed each EEG task only once (i.e., they were not allowed to participate in more than one study included in this dataset).

The final dataset included 127 participants (Mean age: 21.37 years, *SD* = 2.60 years) with sociodemographic and socioeconomic information, and usable data from at least one of the four EEG tasks. Participant reports of gender were as follows: 57.5% female, 41.7% male, and 0.8% cannot be disclosed due to confidentiality concerns. Participants reported their race/ethnicity as follows: 54.3% Hispanic or Latino/x, 18.1% Asian/Asian American, 12.6% selected multiple categories, 12.6% White/European American, 1.6% Black/African American, and 0.8% Middle Eastern. All participants reported having completed at least high school, and 2.4% reported some college credit but less than one year, 19.7% reported one or more years of college but no degree, 10.2% had an associate degree, 18.1% had a bachelor’s degree, and 3.1% had a master’s degree. The highest childhood parental educational attainment levels of the participants were as follows: 24.4% less than a high school diploma, 17.3% high school diploma, 11% some college but no degree, 6.3% associate degree, 19% bachelor’s degree, and 22% professional or graduate degree. Language background information was collected from a subsample of participants (*n* = 82). When asked to report the language they could speak and understand the most fluently, 52 participants reported English, 28 reported Spanish, 2 reported Chinese languages.

### IRB approval

The University of California Merced Institutional Review Board approved the study (IRB #: UCM2022-42). All participants gave written informed consent prior to any data collection. Regarding data sharing permissions and protocols, the consent form informed the participants that (a) after removal of the identifying information, the data could be used for future research studies or distributed to other investigators for future research studies, without additional informed consent from the participant; (b) de-identified data files may be published alongside manuscripts as supplementary information to comply with current data-sharing standards; and (c) de-identified data files will also be deposited to a digital repository.

### Sociodemographic and individual indicators

The dataset includes participant age, gender (open-ended), handedness, and highest level of education. For the highest level of education, participants were asked to select from the categories that were similar to those used in the Survey of Income and Program Participation (SIPP)^31^. Participants were also asked whether they were employed at the time of the study, and if so, whether the employment was full or half-time. For individual and household income, participants were asked to think about all the money they earned over the past 12 months (including money from various jobs, as well other sources of income such as interest from a business, inheritance, earned income tax credits, child support, etc.), and select a category that best described their total income. Participants were also asked to report their household income by considering the total earnings of all adults living with them over the past 12 months. Participant and household income, and participant employment status data were removed from the publicly available dataset^32^ described here due to confidentiality concerns, as explained below. To prevent participants from considering their childhood and adulthood socioeconomic conditions before or during the tasks, we administered the SES questionnaires only after the EEG tasks were completed.

#### Objective childhood family SES

To obtain retrospective reports of objective childhood family SES, we asked participants to think back to when they were 10 years old and answer questions about their parents/legal guardians. Participants could list up to 4 adults, first reporting on the relationship of this adult to them (e.g., mother, father, grandparent, stepparent, etc.), and then the highest level of education completed by this person, and whether this person was living in their household. Educational attainment was reported by selecting one of the provided categories. These categories were designed to align with those used in the SIPP^31^. In a previous publication, these categorical labels were recoded as the highest years of education completed by a parent/legal guardian that the participant reported living with them when they were 10 years old^25^.

While recall biases and inaccuracies can influence self-reported childhood SES, especially reports of family income, research suggests that individuals can recall parental education levels more reliably. For example, when retrospective parental education reports in the Health and Retirement Study were compared with prospective census data from 1940, retrospective reports were found to be highly reliable^33^. Moreover, retrospective parental education measures were found to demonstrate predictive validity comparable to prospective data, supporting their use as an indicator of childhood parent education.

#### Subjective social status in childhood

Subjective social status in childhood was measured with an adapted version of the MacArthur Scale of Subjective Social Status^34^. As with objective childhood family SES, participants were asked to recall their circumstances at age 10 and rank their family’s standing within American society using a ladder scale, with step 1 corresponding to the bottom of the ladder and step 10 corresponding to being at the top.

#### Food security

We used the six-item short form of the United States Department of Agriculture Household Food Security Survey Module^35,36^. Participants were asked to answer questions considering their experiences in the last 12 months.

#### Home and neighborhood characteristics

This survey included 8 items selected from the Adult Wellbeing subsection of the SIPP administered by the United States Census Bureau^31^. The items included questions about where the participants were living over the last month, such as whether there were “holes in the walls or ceiling, or cracks wider than the edge of a dime,” whether “street noise or heavy street traffic” were problems in their neighborhood, and to what extent their neighborhood was safe.

### Language background

A subset of participants completed the Language and Social Background Questionnaire (LSBQ), which assesses the degree of bilingualism in young adults living in communities in which English is the official language^37^. Participants were asked to list all the languages they could speak and understand in order of fluency, including English. Due to the low percentages of some of the languages reported, this information was considered identifiable. Therefore, the first and the second language participants reported were recoded as “English” and “non-English,” consistent with the phrasing used by Anderson and colleagues (2018). In addition to these categorical variables, the present dataset also includes when (i.e., age of acquisition) and where each language was acquired (e.g., home, school, etc.). The researcher who contributed this questionnaire to the study declined to share the rest of the data collected as part of the LSBQ.

#### Attention-Deficit/Hyperactivity Disorder (ADHD) symptoms

The World Health Organization (WHO) Adult ADHD Self-Report Scale (ASRS-V1.1)^30^ was used to assess symptoms consistent with ADHD in adults. This scale consists of 18 DSM-IV-TR criteria. The first 6 questions (considered Part A in the original screener) have been considered the most predictive of symptoms consistent with ADHD^30^. However, to provide a more comprehensive account of ADHD-like symptoms in adulthood, we administered and hereby share the responses to all items. Participants were asked to rate themselves on each criterion by selecting the category (never, rarely, sometimes, often, very often) that best described how they felt and conducted themselves over the past 6 months. We recoded the participant responses to the first 6 items (Part A of the scale) into dichotomous variables (0/1) as described in the manual of this self-report scale. In addition, following the procedure described in the ASRS-V1.1, the total scores across these 6 dichotomous variables were used to create a binary variable reflecting whether the participant has “symptoms highly consistent with ADHD in adults.” The dataset includes the raw item-level data (responses to all questions), the dichotomized Part A items, and the binary indicator of symptoms consistent with ADHD in adults.

### EEG recording

**Fig. 1:**
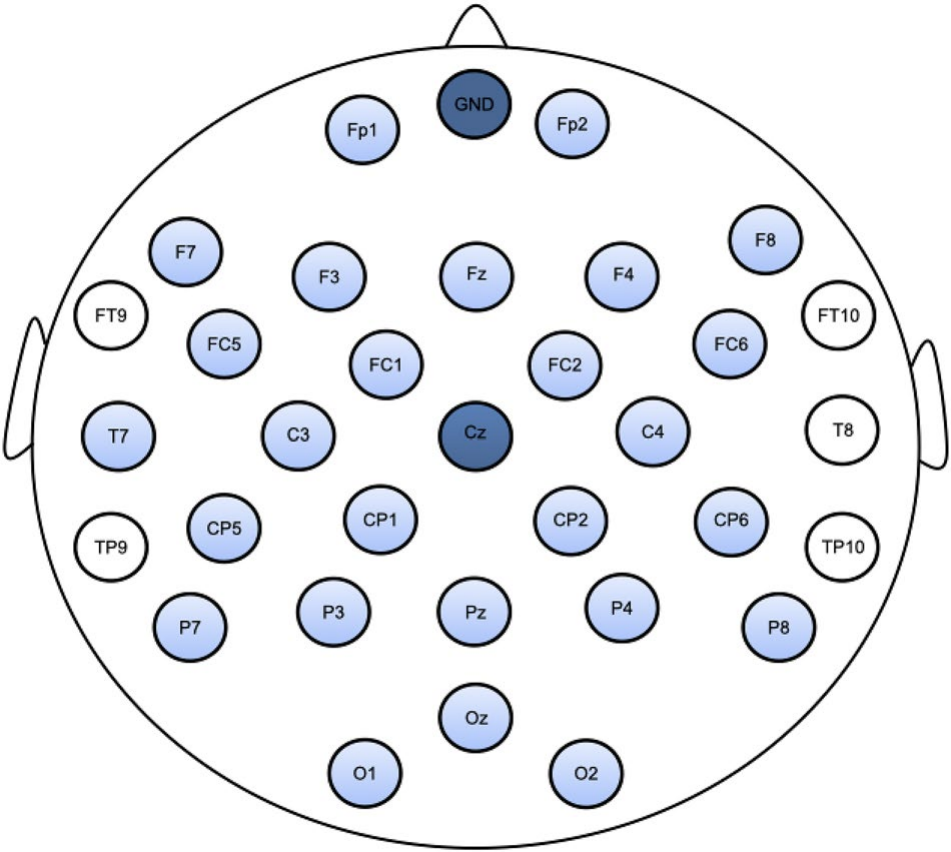
Electrode configuration. 32-channel EEG electrode system with 10/20 electrode configuration. The ground electrode (placed at the location of FPz and marked as GND) and the online reference electrode (Cz) are shown in dark blue. The electrodes that were pulled from the bundle and repurposed as mastoid and EOG electrodes are shown in white. The scalp electrodes included in EEG recordings are shaded in light blue. The mastoid and the EOG electrodes are not depicted.

EEG data were recorded using the Brain Products actiCHamp Plus system, in combination with BrainVision Recorder (Version 1.25.0101). A 32-channel actiCAP slim active electrode system was used, with electrodes mounted on elastic snap caps (Brain Products GmbH, Gilching, Germany). The ground electrode was placed at FPz. From the electrode bundle, 2 electrodes were repurposed and placed on the mastoid bones behind the left and right ears to be used for re-referencing after data collection. To record electrooculogram (EOG), we repurposed 3 additional electrodes. To capture eye artifacts, we placed one horizontal EOG (HEOG) electrode lateral to the external canthus of each eye and one vertical EOG (VEOG) electrode below the right eye. We used the remaining 27 electrodes as scalp electrodes, which were mounted per the international 10/20 system. Figure 1 depicts the electrode configuration. EEG data were recorded at a sampling rate of 500 Hz and referenced to the Cz electrode.

EEG files were recorded for each task separately. StimTrak was used to assess stimulus presentation delays for the monitor and the headphones. The results indicated that both the visual and auditory stimuli had a delay of approximately 20 milliseconds. Therefore, EEG event codes should be shifted in time accordingly.

### EEG tasks

The active visual oddball, flanker, and visual search tasks were directly adopted from the ERP CORE task battery^1^. The passive auditory oddball task was a modified version of the original ERP CORE task^25^. The tasks were administered using the Presentation software from Neurobehavioral Systems. The auditory stimuli were presented via Bose over-ear noise-canceling headphones. Visual stimuli were displayed on a Dell LCD monitor with a 1280 × 1024 resolution, a 60 Hz refresh rate, and a viewing distance of 65 cm. Participants were seated on a comfortable chair in a sound-attenuated recording booth and were offered to take a break in between tasks. In each visual task, there was a white fixation point (0.15° visual angle) at the center of the screen. Participants were asked to keep looking at this point throughout the task. We gave participants verbal instructions at the beginning of each task, which were followed by written instructions displayed on the screen. A Logitech game controller was used to collect behavioral responses.

#### Passive auditory oddball

The original ERP CORE passive auditory oddball task, consisting of 1,000 trials (~10 minutes), was modified for two main reasons. First, in pilot testing, we observed participants getting sleepy as the “silent video” that accompanied the auditory tones had progressed. Therefore, we decided to reduce the duration of the task. Second, we wanted to have an auditory oddball task we could use with a wider age range, especially with young children, in other research projects in the lab. Therefore, we shortened the task duration (approximately 3.5 minutes long) and replaced the video with a child-friendly cartoon, “Pingu” the penguin. As changes to both stimuli properties and the interstimulus interval can influence the mismatch negativity (MMN)^38,39^, we avoided any other changes to the original ERP CORE^1^ task (i.e., the ratio of frequent to rare tones and the tones themselves were kept constant).

In this task, the frequent (standard) tone appeared in 80% of trials, presented at 80 dB for 100 milliseconds. The rare (oddball) tone occurred in the remaining 20% of trials, presented at 70 dB for 100 milliseconds. Interstimulus intervals jittered between 450–550 milliseconds. To allow the auditory system to habituate to the frequent tone, the task began with 15 frequent tones. Participants were informed that they would hear sounds through the headphones and were instructed to disregard them while watching a silent video presented on the computer monitor. Participants were presented with a total of 350 trials.

The ERP CORE passive auditory oddball task was originally designed to elicit the MMN ERP component^1^. To ensure the modified version we used in the present dataset elicited a robust MMN, we examined the MMN elicited in the original ERP CORE sample based on our data processing pipeline, the MMN elicited in a pilot sample whose data was collected in our lab using the exact same ERP CORE task, and the MMN elicited in the task modified by our lab. Similar to the ERP CORE task, the modified version used in our dataset also successfully elicited a robust MMN component, as detailed in a previous publication^25^.

#### Active visual oddball

On each trial, a single capitalized letter (A, B, C, D, or E) was presented. One letter was the target for a given block (20% of trials, i.e., rare/oddball stimuli), and the other 4 letters were non-targets (80% of trials, i.e., frequent/standard stimuli). The visual stimuli were displayed against a medium gray background (x = 0.35, y = 0.36, 25.9 cd/m^2^). The letters were presented in the Geneva font and covered a visual angle of 2.5 × 2.5°. Each letter was presented for 200 milliseconds. The interstimulus interval was 1200–1400 milliseconds. Participants were instructed to indicate whether the stimulus presented was a target or non-target, using the “up” button press for targets and the “down” button press for non-targets, on the game controller. The task consisted of 200 trials, divided into 5 blocks. Each letter functioned as a target in one experimental block and as a non-target in the remaining 4 blocks. Block order was randomized for each participant.

#### Simple visual search

At the beginning of each block, participants were given a target color (pink or blue). On each trial, 24 outlined squares (0.45 × 0.45°) with a gap (0.3° visual angle) on one side were presented in the left and right visual fields (i.e., 12 items per visual field). In each visual field, there were 11 black squares with a gap opening to the left or right side, determined randomly and independently. In addition, either 1 blue (x = 0.28, y = 0.33, 116 cd/m2) or 1 pink square (x = 0.35, y = 0.30, 118 cd/m2), with a gap on either the top or the bottom, was presented in each visual field. The blue and pink squares were always presented in opposite visual fields. Their location was randomized across trials. Each stimuli array was presented for 500 milliseconds. The interstimulus interval was 900–1100 milliseconds. The target was either the blue or the pink square (0.50 probability) and the order of the targets was counterbalanced across participants. Participants were instructed to indicate whether the target square had a top or bottom gap, using the “up” button press when the target had a top gap and the “down” button press when the target had a bottom gap, on the game controller. The task consisted of 320 trials, divided into 8 blocks.

Although each participant was instructed at the beginning of the task to maintain fixation to the fixation point and the fixation point was visible throughout the task, we observed notable amounts of saccades for several participants after data collection. This implies a potential breach in data collection protocol for some participants, where the experimenter did not regularly warn the participants who were moving their eyes from the fixation point to the visual field where the target was located. Therefore, regardless of whether an artifact correction or artifact rejection method is used, user discretion is advised for the rejection of trials or exclusion of participants with notable saccades.

#### Flanker

On each trial, a horizontal array of 5 arrowheads was presented, centered on a fixation point that was continuously visible throughout the task. Each arrowhead (1 × 1° visual angle) pointed either left or right, with the central arrowhead always serving as the target. In half of the trials, the flanking arrowheads matched the target’s direction (congruent), while in the other half, they pointed in the opposite direction (incongruent). Each array was presented for 200 milliseconds. The interstimulus interval was 1200–1400 milliseconds. The combinations of target direction (left vs. right) and flanker type (congruent vs. incongruent) were randomized. Participants were instructed to press a button with their left or right hand to match the target’s direction. The task consisted of 400 trials, divided into 10 blocks. The task was optimized to elicit error-related neural responses^1^. Therefore, to ensure a reasonable amount of error rate, participants received feedback at the end of each block as follows: If the error rate fell below 10%, participants were shown the message: “Try to respond a bit faster.” If the error rate exceeded 20%, participants were shown the message: “Try to respond more accurately.”

### De-identification of data

All data were labeled with unique participant identifiers (ID). Before the data were publicly shared, all identifiable information was removed, including date of birth, date of testing, race/ethnicity, zip code, occupation (of self and parents/legal guardians), income (self and household), and names of the languages the participants reported speaking and understanding fluently. Date of birth was used to compute age in years. The summary of race/ethnicity and language background information is reported in the Participants section.

After the removal of these variables, we performed formal re-identification risk assessments using the statistical disclosure control for micro-data (*sdcMicro*) package in R^40^. Following the results of an initial risk assessment, several quasi-identifiers were grouped based on logical thresholds and distribution characteristics. Age was binned into three categories (18 to 22, 23 to 26, and 27 to 30). Highest level of education attained by the participant and a parent was grouped into three categories: (1) “High School or Less” (including “Less than a high school diploma” and “High school diploma”), (2) “Some College or Associate Degree” (including “Some college, no degree” and “Associate degree”), and (3) “Bachelor’s Degree or Higher” (including “Bachelor’s degree” and “Professional or graduate degree”). These groupings were designed to preserve interpretability while minimizing the number of unique combinations that could lead to participant re-identification.

Furthermore, to prepare the dataset for public sharing, rare responses were masked as “cannot be disclosed,” and one quasi-identifier (employment status) was removed after it was found to contribute significantly to participant uniqueness. With these strategies, the global re-identification risk was reduced to 17%. The global risk achieved here reflects a balance between protecting confidentiality, preserving analytic utility, and meeting BIDS standards.

## Data Records

**Fig. 2:**
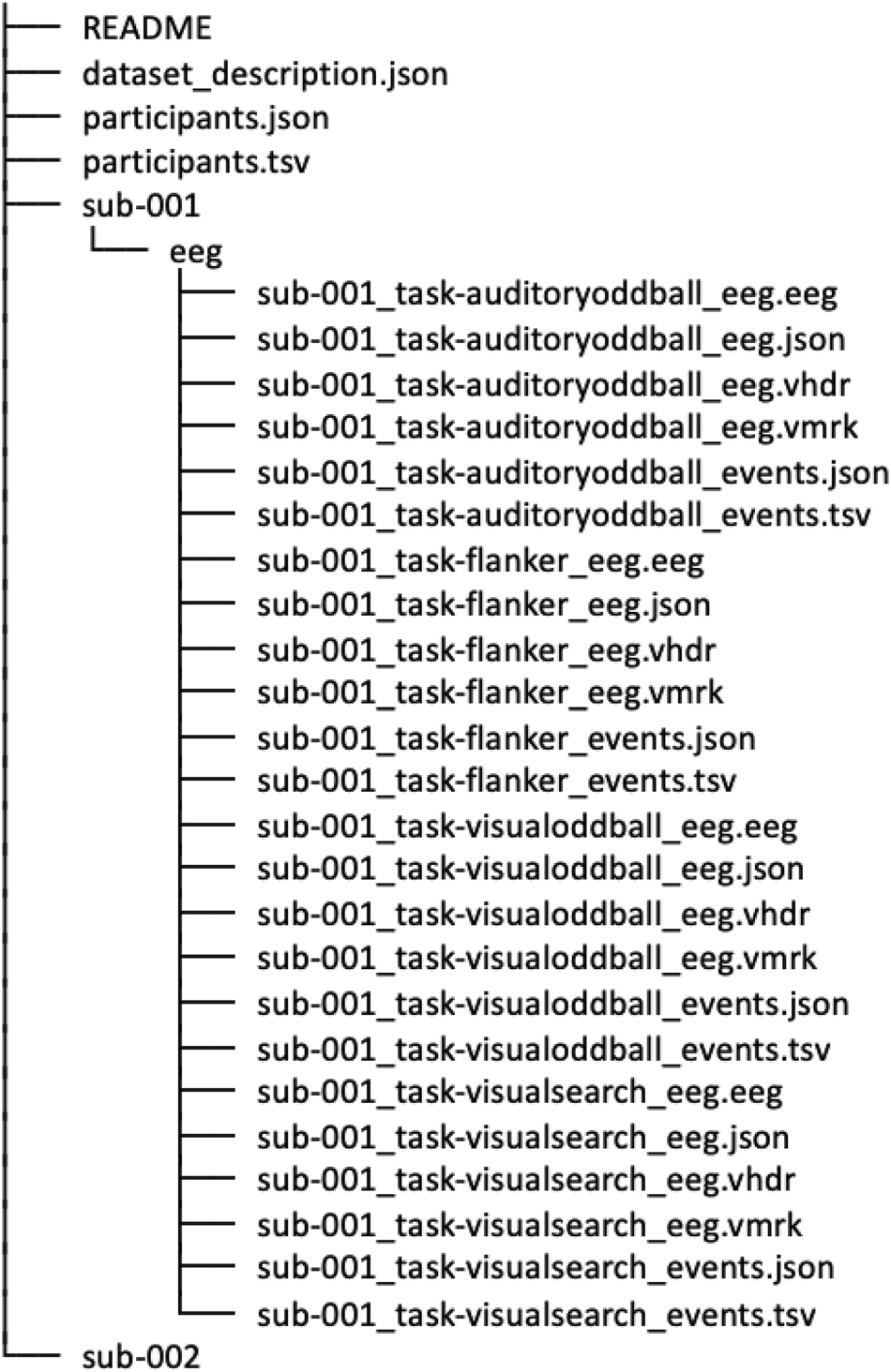
The structure of the dataset in BIDS format.

The dataset is available on OpenNeuro^32^, in accordance with the Brain Imaging Data Structure (BIDS) guidelines^41,42^. The main folder of the dataset on OpenNeuro contains 127 subfolders, one for each participant, and 4 additional files: (i) “dataset_description.json” that provides information on the dataset and registration details; (ii) “participants.tsv” that contains sociodemographic information about the participants, such as age and gender, as well as participant responses from the self-report measures mentioned above; (iii) “participants.json” that describes all of the columns presented in the “participants.tsv” file; and (iv) “README” that provides general information about the dataset, including contact information. Each participant’s folder contains the EEG data and event information for each of the EEG tasks they completed. The data structure is shown in Fig. 2.

## Technical Validation

To ensure high quality EEG data collection, trained researchers constantly monitored EEG waves on a separate monitor during the entire session. If any issues that could be addressed were observed (e.g., excessive participant movement or eye artifacts, excessive channel drift), the researcher addressed these issues during task breaks to avoid distracting the participant. If any issues were observed during data collection and could not be easily resolved (e.g., a problem with a scalp electrode, a technical issue), these observations were recorded in a data collection log. These observations are shared as a part of the dataset.

To further document any EEG channel issues, we examined which channels contained excessive noise (i.e., “bad” channels). First, we applied a band-pass filter from 0.1 Hz to 40 Hz using the EEGLAB default finite impulse response (FIR) filter (−6 dB cutoff frequency). Then, we used EEGLAB’s *pop_rejchan.m* function^43^. To provide a stricter threshold, we lowered the default threshold from 5 to 3 standard deviations. Given that the channels that are used for artifact detection are by default noisier and can obscure the presence of otherwise noisy scalp channels, we limited these outlier analyses to the scalp channels. The EEG scalp channels that were marked as noisy are reported in the dataset separately for each task, with variables providing a total number of noisy channels per participant per task, as well as a record of the name of the noisy channels per task. Most participants had 0–2 noisy scalp channels, and no participant had more than 3 noisy scalp channels. A summary of the percentage of participants with noisy channels per task is provided in Table 1.

**Table 1:** Percentage of participants with zero, one, two, or three noisy channels per task.

Task	Zero	One	Two	Three
Auditory Oddball	57.90	39.47	2.63	0.00
Visual Oddball	60.20	34.70	5.10	0.00
Visual Search	63.89	30.56	5.55	0.00
Flanker	53.42	36.99	8.22	1.37

Before depositing the data, we visually inspected each EEG data file to ensure none were compromised. We made all EEG data available to users regardless of our own subjective ratings of data quality. The decision to share all the data regardless of quality ratings was based on three factors. First, the users may have different needs and may choose to opt for visual inspection of continuous data and/or use objective data quality measures, such as indicators of precision or reliability^44,45^. Second, with rapid advances in data processing procedures, what we may consider low data quality now may become acceptable in the future with different artifact correction methods. Third, depending on researcher interests, with access to various participant characteristics, individual differences in data quality as well as whether missingness is at random within and across tasks can be examined.

As per technical validation of the data structure, OpenNeuro BIDS validator was used before the dataset was uploaded to OpenNeuro and the dataset was confirmed as “valid” before release.

### Sampling biases and representativeness

To ensure recruitment of participants from diverse socioeconomic backgrounds, we posted flyers in community centers, local businesses, and public spaces, such as hospitals, local bookstores, and coffee shops. We selected these locations by requesting permission to post flyers and placed flyers at all locations where we received approval. Regardless of the recruitment efforts, the majority of our participants were undergraduate students. Our self-report measures only asked about participant occupation and did not ask about whether the participant was a student or not. While 61% of participants explicitly reported being undergraduate students in our sociodemographic survey, this figure is likely an underestimation. We suspect that those who held part-time or full-time jobs may have reported their occupation rather than their student status. Therefore, while our recruitment strategy aimed to reach a socioeconomically diverse young adult population, the resulting sample likely overrepresents individuals with some connection to higher education, a limitation to broader generalizability that should be addressed through alternative recruitment strategies.

Having acknowledged this, it is also important to note that the university where data collection took place is unique in its student composition. It is the only public research university in the United States where over 60% of students qualify for a federal financial aid program awarded to undergraduates with demonstrated financial need in the United States. Furthermore, approximately 65% of students at this institution are reported to be the first in their families to attend college. Although our sample includes a high proportion of students, these institutional characteristics suggest that these participants were likely to be sampled from a population of students from lower-income and lower-parent-education backgrounds. Furthermore, as reported above, only 41% of the participants in our sample reported that their parents/legal guardians had obtained a bachelor’s degree or higher. These characteristics suggest that, despite the acknowledged overrepresentation of individuals with some connection to higher education, our sample includes individuals from family socioeconomic backgrounds that likely have been underrepresented in cognitive neuroscience research, even within higher education settings.

## Usage Notes

We decided to share this dataset in its entirety soon after the data collection permanently stopped for these projects with adult participants, in accordance with the principles of open data dissemination. As noted above, any analyses that are time-locked to the visual and auditory stimuli should be conducted after the event codes are shifted to reflect the 20-millisecond delay due to the monitor and the headphones. Questions regarding this dataset can be directed to the corresponding authors or posted as a comment on the dataset’s OpenNeuro.org page.

## Data Availability

The EEG tasks were originally optimized to capture ERP components of interest. The ERP CORE experimental tasks are publicly available on Open Science Framework^46^, under Experiment Control Files. Examples of data processing codes for the ERP CORE tasks can also be found on Open Science Framework^46,47^.

## References

[1] Kappenman, E. S., Farrens, J. L., Zhang, W., Stewart, A. X. & Luck, S. J. ERP CORE: An open resource for human event-related potential research. *Neuroimage* **225**, 117465 (2021). 33099010 10.1016/j.neuroimage.2020.117465 PMC7909723

[2] Cohen, M. X. Where Does EEG Come From and What Does It Mean? *Trends Neurosci.* **40**, 208–218 (2017). 28314445 10.1016/j.tins.2017.02.004

[3] Bastiaansen, M., Mazaheri, A. & Jensen, O. Beyond ERPs: Oscillatory Neuronal Dynamics. in *The Oxford Handbook of Event-Related Potential Components* (eds. Kappenman, E. S. & Luck, S. J.) 32–50, 10.1093/oxfordhb/9780195374148.013.0024 (Oxford University Press, 2011).

[4] Luck, S. J. *et al*. Electroencephalography and Event-Related Brain Potentials. in *Handbook of Psychophysiology* 74–100. 10.1017/9781107415782.005 (Cambridge University Press, 2016).

[5] Buzsáki, G. *Rhythms of the Brain*. (Oxford University Press, Oxford, England, 2006).

[6] Cohen, M. X. *Analyzing Neural Time Series Data: Theory and Practice*. (Mit Press, 2014).

[7] O’Donnell, B. F., Salisbury, D. F., Niznikiewicz, M. A., Brenner, C. A. & Vohs, J. L. Abnormalities of Event-Related Potential Components in Schizophrenia. in *The Oxford Handbook of Event-Related Potential Components* (eds. Kappenman, E. S. & Luck, S. J.) **0**, 10.1093/oxfordhb/9780195374148.013.0251 (Oxford University Press, 2011).

[8] Bruder, G. E., Kayser, J. & Tenke, C. E. Event-Related Brain Potentials in Depression: Clinical, Cognitive, and Neurophysiological Implications. in *The Oxford Handbook of Event-Related Potential Components* (eds. Kappenman, E. S. & Luck, S. J.) 564–592, 10.1093/oxfordhb/9780195374148.013.0257 (Oxford University Press, 2011).

[9] Luck, S. J. *et al*. Impaired response selection in schizophrenia: evidence from the P3 wave and the lateralized readiness potential. *Psychophysiology* **46**, 776–786 (2009). 19386044 10.1111/j.1469-8986.2009.00817.x PMC2706937

[10] Arns, M., Conners, C. K. & Kraemer, H. C. A decade of EEG theta/beta ratio research in ADHD: a meta-analysis. *J. Atten. Disord.* **17**, 374–383 (2013). 23086616 10.1177/1087054712460087

[11] Michelini, G., Salmastyan, G., Vera, J. D. & Lenartowicz, A. Event-related brain oscillations in attention-deficit/hyperactivity disorder (ADHD): a systematic review and meta-analysis. *Int. J. Psychophysiol.* **174**, 29–42 (2022). 35124111 10.1016/j.ijpsycho.2022.01.014 PMC13094451

[12] O’Reilly, C., Lewis, J. D. & Elsabbagh, M. Is functional brain connectivity atypical in autism? A systematic review of EEG and MEG studies. *PLoS One* **12**, e0175870 (2017). 28467487 10.1371/journal.pone.0175870 PMC5414938

[13] V. M. Dotson, V. M. Dotson & A. Duarte. The importance of diversity in cognitive neuroscience. *Ann. N. Y. Acad. Sci*. 10.1111/nyas.14268 (2020). 10.1111/nyas.14268 31663150

[14] Prather, R. W. A new path: Why we need critical approaches to cognitive and psychological sciences. *J. Appl. Res. Mem. Cogn.* **12**, 195–198 (2023).

[15] Kissel, H. A. & Friedman, B. H. Participant diversity in Psychophysiology. *Psychophysiology* **60**, e14369 (2023). 37332087 10.1111/psyp.14369

[16] Noble, K. G. & Giebler, M. A. The neuroscience of socioeconomic inequality. *Curr Opin Behav Sci* **36**, 23–28 (2020). 32719820 10.1016/j.cobeha.2020.05.007 PMC7384696

[17] Rakesh, D. & Whittle, S. Socioeconomic status and the developing brain - A systematic review of neuroimaging findings in youth. *Neurosci. Biobehav. Rev.* **130**, 379–407 (2021). 34474050 10.1016/j.neubiorev.2021.08.027

[18] Olson, L., Chen, B. & Fishman, I. Neural correlates of socioeconomic status in early childhood: a systematic review of the literature. *Child Neuropsychol.* **27**, 390–423 (2021). 33563106 10.1080/09297049.2021.1879766 PMC7969442

[19] Stevens, C., Lauinger, B. & Neville, H. Differences in the neural mechanisms of selective attention in children from different socioeconomic backgrounds: an event-related brain potential study. *Dev. Sci.* **12**, 634–646 (2009). 19635089 10.1111/j.1467-7687.2009.00807.x PMC2718768

[20] Hampton Wray, A. *et al*. Development of selective attention in preschool-age children from lower socioeconomic status backgrounds. *Dev. Cogn. Neurosci.* **26**, 101–111 (2017). 28735165 10.1016/j.dcn.2017.06.006 PMC5703215

[21] St John, A. M., Finch, K. & Tarullo, A. R. Socioeconomic status and neural processing of a go/no-go task in preschoolers: An assessment of the P3b. *Dev. Cogn. Neurosci*. 100677 (2019). 10.1016/j.dcn.2019.100677 PMC6969333 31255904

[22] Peters, A., Zeytinoglu, S., Leerkes, E. M. & Isbell, E. Component-specific developmental trajectories of ERP indices of cognitive control in early childhood. *Dev. Cogn. Neurosci.* **64**, 101319 (2023). 37907010 10.1016/j.dcn.2023.101319 PMC10632416

[23] Dufford, A. J., Evans, G. W., Liberzon, I., Swain, J. E. & Kim, P. Childhood socioeconomic status is prospectively associated with surface morphometry in adulthood. *Dev. Psychobiol.* **63**, 1589–1596 (2021). 33432574 10.1002/dev.22096 PMC13102113

[24] Javanbakht, A. *et al*. Childhood poverty predicts adult amygdala and frontal activity and connectivity in response to emotional faces. *Front. Behav. Neurosci.* **9**, 154 (2015). 26124712 10.3389/fnbeh.2015.00154 PMC4464202

[25] Isbell, E., De León, N. E. R. & Richardson, D. M. Childhood family socioeconomic status is linked to adult brain electrophysiology. *PLoS One* **19**, e0307406 (2024). 39163384 10.1371/journal.pone.0307406 PMC11335154

[26] Loued-Khenissi, L. *et al*. Signatures of life course socioeconomic conditions in brain anatomy. *Hum. Brain Mapp.* **43**, 2582–2606 (2022). 35195323 10.1002/hbm.25807 PMC9057097

[27] M. Y. Chan *et al*. Socioeconomic status moderates age-related differences in the brain’s functional network organization and anatomy across the adult lifespan. *Proc. Natl. Acad. Sci. USA*10.1073/pnas.1714021115 (2018). 10.1073/pnas.1714021115 PMC5984486 29760066

[28] Yaple, Z. A. & Yu, R. Functional and Structural Brain Correlates of Socioeconomic Status. *Cereb. Cortex* **30**, 181–196 (2020). 31044253 10.1093/cercor/bhz080

[29] Muscatell, K. A. *et al*. Neural mechanisms linking social status and inflammatory responses to social stress. *Soc. Cogn. Affect. Neurosci.* **11**, 915–922 (2016). 26979965 10.1093/scan/nsw025 PMC4884319

[30] Kessler, R. C. *et al*. The World Health Organization Adult ADHD Self-Report Scale (ASRS): a short screening scale for use in the general population. *Psychol. Med.* **35**, 245–256 (2005). 15841682 10.1017/s0033291704002892

[31] United States Census Bureau. Survey of Income and Program Participation (SIPP). https://www.census.gov/programs-surveys/sipp.html (2022).

[32] Isbell, E., Peters, A. N., Richardson, D. M. & De León, N. E. R. Cognitive Electrophysiology in Socioeconomic Context in Adulthood: An EEG dataset - *OpenNeuro*. 10.18112/openneuro.ds006018.v1.2.1 (2025). 10.1038/s41597-025-05209-z PMC12098793 40404753

[33] Warren, J. R., Lee, M. & Osypuk, T. L. The validity and reliability of retrospective measures of childhood socioeconomic status in the Health and Retirement Study: Evidence from the 1940 U.s. census. *J. Gerontol. B Psychol. Sci. Soc. Sci.* **77**, 1661–1673 (2022). 35263760 10.1093/geronb/gbac045 PMC9434433

[34] Adler, N. E., Epel, E. S., Castellazzo, G. & Ickovics, J. R. Relationship of subjective and objective social status with psychological and physiological functioning: preliminary data in healthy white women. *Health Psychol.* **19**, 586–592 (2000). 11129362 10.1037//0278-6133.19.6.586

[35] Blumberg, S. J., Bialostosky, K., Hamilton, W. L. & Briefel, R. R. The effectiveness of a short form of the Household Food Security Scale. *Am. J. Public Health* **89**, 1231–1234 (1999). 10432912 10.2105/ajph.89.8.1231 PMC1508674

[36] Rabbitt, M. P., Hales, L. J. & Reed-Jones, M. Six-Item Short Form of the Food Security Survey Module. *Food Security in the U.S. - Survey Tools* https://www.ers.usda.gov/topics/food-nutrition-assistance/food-security-in-the-us/survey-tools#six (2012).

[37] Anderson, J. A. E., Mak, L., Keyvani Chahi, A. & Bialystok, E. The language and social background questionnaire: Assessing degree of bilingualism in a diverse population. *Behav. Res. Methods* **50**, 250–263 (2018). 28281208 10.3758/s13428-017-0867-9 PMC5591752

[38] Näätänen, R., Pakarinen, S., Rinne, T. & Takegata, R. The mismatch negativity (MMN): towards the optimal paradigm. *Clin. Neurophysiol.* **115**, 140–144 (2004). 14706481 10.1016/j.clinph.2003.04.001

[39] Näätänen, R., Kujala, T. & Light, G. *Mismatch Negativity: A Window to the Brain*. (Oxford University Press, 2019).

[40] Templ, M., Kowarik, A. & Meindl, B. Statistical disclosure control for micro-data using theRPackagesdcMicro. *J. Stat. Softw.* **67**, 1–36 (2015).

[41] Gorgolewski, K. J. *et al*. The brain imaging data structure, a format for organizing and describing outputs of neuroimaging experiments. *Sci Data* **3**, 160044 (2016). 27326542 10.1038/sdata.2016.44 PMC4978148

[42] Pernet, C. R. *et al*. EEG-BIDS, an extension to the brain imaging data structure for electroencephalography. *Sci Data* **6**, 103 (2019). 31239435 10.1038/s41597-019-0104-8 PMC6592877

[43] Delorme, A. & Makeig, S. EEGLAB: an open source toolbox for analysis of single-trial EEG dynamics including independent component analysis. *J. Neurosci. Methods* **134**, 9–21 (2004). 15102499 10.1016/j.jneumeth.2003.10.009

[44] Luck, S. J., Stewart, A. X., Simmons, A. M. & Rhemtulla, M. Standardized measurement error: A universal metric of data quality for averaged event-related potentials. *Psychophysiology* e13793, 10.1111/psyp.13793 (2021). 10.1111/psyp.13793 PMC8169536 33782996

[45] Clayson, P. E., Carbine, K. A., Baldwin, S. A., Olsen, J. A. & Larson, M. J. Using generalizability theory and the ERP Reliability Analysis (ERA) Toolbox for assessing test-retest reliability of ERP scores part 1: Algorithms, framework, and implementation. *Int. J. Psychophysiol*. 10.1016/j.ijpsycho.2021.01.006 (2021). 10.1016/j.ijpsycho.2021.01.006 33465427

[46] Kappenman, E. S., Farrens, J., Zhang, W., Stewart, A. X. & Luck, S. J. ERP CORE. https://osf.io/thsqg (2020). 10.1016/j.neuroimage.2020.117465 PMC7909723 33099010

[47] Isbell, E., De León, N. E. R. & Richardson, D. M. Childhood family socioeconomic status is linked to adult brain electrophysiology - accompanying analytic data and code. https://osf.io/43h75/ (2024). 10.1371/journal.pone.0307406 PMC11335154 39163384

